# Microfluidic Technology, Artificial Intelligence, and Biosensors As Advanced Technologies in Cancer Screening: A Review Article

**DOI:** 10.7759/cureus.39634

**Published:** 2023-05-29

**Authors:** Jawad Noor, Ahtshamullah Chaudhry, Saima Batool

**Affiliations:** 1 Internal Medicine, St. Dominic Hospital, Jackson, USA; 2 Pathology, Nishtar Medical University, Multan, PAK

**Keywords:** advancements, biosensors, artificial intelligence, microfluidic technology, cancer screening

## Abstract

Cancer screening techniques aim to detect premalignant lesions and enable early intervention to delay the onset of cancer while keeping incidence constant. Technology advancements have led to the development of powerful tools such as microfluidic technology, artificial intelligence, machine learning algorithms, and electrochemical biosensors to aid in early cancer detection. Non-invasive cancer screening methods like virtual colonoscopy and endoscopic ultrasonography have also been developed to provide comprehensive pictures of organs and detect cancer early. This review article provides an overview of recent advances in cancer screening in microfluidic technology, artificial intelligence, and biomarkers through a narrative literature search. Microfluidic devices enable easy handling of sub-microliter volumes and have become a promising tool for cancer detection, drug screening, and modeling angiogenesis and metastasis in cancer research. Machine learning and artificial intelligence have shown high accuracy in oncology-related diagnostic imaging, reducing the manual steps in lesion detection and providing standardized and accurate results, with potential for global standardization in areas like colon polyps, breast cancer, and primary and metastatic brain cancer. A biomarker-based cancer diagnosis is promising for early detection and effective therapy, and electrochemical biosensors integrated with nanoparticles offer multiplexing and amplification capabilities. Understanding these advanced technologies' basics, achievements, and challenges is crucial for advancing their use in oncology.

## Introduction and background

Cancer is second to heart disease as the leading cause of death in the United States [1]. Cancer screening is a technique for preventing cancer at an early stage. According to Centers for Disease Control and Prevention (CDC) data, in 2019 total of 1,752,735 new cases of cancer were reported, with 599,589 deaths in the United States. It is important to note that the incident data of 2019 is the most recent data available. The good news is that screening may help detect certain cancers, including colon, lung, cervical, and breast cancer, which can help to slow down or even halt the progression of the illness [1]. Screening is a technique for a secondary level of prevention that aims to decrease cancer mortality while maintaining the incident rate constant. Given a significant lag time in the malignant transition, screening presents an opportunity to detect premalignant lesions, engage in early intervention in the carcinogenic process, and delay the onset of cancer [2].

Advances in cancer screening technologies and methods have been made possible by the rapid progress of science and technology. For example, Microfluidic technology allows the manipulation of fluids on a micron scale, making it a powerful tool for diagnosing cancer [3]. Similarly, Artificial Intelligence and machine learning algorithms have been applied to medical data, enabling faster and more accurate identification of cancer risk factors and early cancer detection [4]. Electrochemical biosensors, which can be divided into immunosensors, apt sensors, enzymatic biosensors, and nucleic acid biosensors, are preferred due to their sensitivity, specificity, affordability, and potential for miniaturization in cancer biomarker detection [5].

The development of non-invasive cancer screening methods has significantly advanced cancer screening. For example, to obtain comprehensive pictures of the colon and enable the early identification of colon cancer, a non-invasive procedure called virtual colonoscopy is used [6]. Similar to MRIs, endoscopic ultrasonography employs sound waves to provide fine-grained pictures of inside organs like the pancreas, making it possible to detect pancreatic cancer early [7].

This review article will provide an overview of the recent advances in cancer screening, including the development of microfluidic technology, artificial intelligence, and biomarkers. We will discuss the benefits and limitations of these advances and their potential impact on cancer prevention and management. This review aims to provide a comprehensive summary of the current state of cancer screening and to highlight the ongoing research and future directions in this important area of cancer care.

## Review

Methodology

This is a narrative review where the authors have conducted extensive literature searches using online databases such as Google Scholar, PubMed, Sci-Hub, Elsevier, and others to gather information from previously published articles. The goal of this literature review was to collect information from previously published articles that are relevant to the topic under discussion.

To ensure a comprehensive review, the authors used various search terms like: "Advances in Cancer Screening", "Microfluidic Technology", "Artificial Intelligence", and "Biosensors". The selected articles were carefully reviewed and analyzed to extract essential trends and insights about the topic under discussion.

Microfluidic technology

Microfluidic technology allows the manipulation of fluids on a micron scale, making it a powerful tool for diagnosing cancer. It can perform complex laboratory techniques on a microchip with high precision and efficiency, allowing scientists to demonstrate that their platform was a reliable and simple method to isolate single cells without any labeling, which is crucial for the research of particular therapeutic target detection of cancer cells and cell-derived products [8]. The technology can also be used for therapeutic target discovery and treatment response monitoring. Microfluidic devices can extract individual tumor cells, allowing for genomics, transcriptomics, and metabolomics analysis. It can capture unusual cells, including circulating stem cells, fetal cells, and tumor cells. The platform is also useful for investigating specific processes and treatment options for metastases, which cause more than 90% of cancer deaths [9]. A drawback of microfluidic technology is the absence of an appropriate microfluidic device that can carry out every laboratory process on a single chip [10]. Nevertheless, by decreasing and removing the barriers to accurate detection, the technology has the ability to detect cancer early on and save lives.

Microfluidic systems provide a substantial opportunity to use sensor devices for various applications, including clinical diagnostics, biological detection, and environmental or wastewater monitoring. In order to track the movement of a single cancer cell, Nguyen et al. devised an electrical cell-impedance sensing system that was combined with a microfluidic device [11]. Without the need for physical contact like off chip pneumatics, microfluidics approaches may effectively collect cancer cells consecutively. The identification of single cancer cell migration was successfully shown using the real-time detection of MDA MB 231 cells in the early migration phases in metastases [11]. This method may be used as a cutting-edge tool in studying cancer cells.

Shah et al. put into practice a clever technique for cell recovery from microfluidic devices employing a biopolymer system. A complicated substance, such as tumor cells in whole blood, is isolated specifically utilizing an affinity-based microfluidic device. They used this method to capture and release cancer cells that expressed the epithelial cell adhesion molecule (EpCAM) [12]. The process involves exposing cancer cells that express Ep-CAM to a gel functionalized with anti-Ep-CAM molecules. The gel is then dissolved to release the cancer cells. Cell capture, release, and recovery rates were used to measure the efficiency of the cell release process. The viability of the release cells was determined to be 98.9%±0.3% compared to the viability of control cells, which was 99.4%±0.6%. The liberated cells were then tested for their ability to proliferate by diluting them in a culture medium. At 96 hours, the data showed a proliferation capacity of 69.3%±3.4, compared to a control value of 68.8%±2.2 [12].

Rather than the primary tumors, metastasis, and its associated complications are the leading cause of most cancer-related deaths. Zhao et al. developed microfluidic devices to merge tumor and stromal cell spheroids in a controlled setting to replicate the process of metastasis in a 3-D metastatic analysis [13]. This research aimed to evaluate the metastatic process of tumor-stromal cell spheroids in a three-dimensional (3D) cell culture system using microwell array microfluidic devices. Traditional 3D culture methods are incapable of precisely pairing and quantifying the coexistence of diverse cell populations. The researchers used microfluidic devices to produce accurate one-to-one pairing of tumor and fibroblast spheroids, allowing them to analyze 3D tumor invasion using high-content imaging [13].

Chu et al. created an attomolar-level multiplex-microRNA (miRNA) detection system for cancer diagnostics employing nanomaterial-based microfluidic biochips [14]. The assembly utilized graphene oxide (GO) as a special nanomaterial. The scientists used five miRNA biomarkers (miR-125, miR-126, miR-191, miR-155, and miR-21) often present in breast cancer to assess the effectiveness of their microfluidic device [14]. The microfluidic biochip detected these miRNA markers accurately in breast cancer samples, with a detection time of around 35 minutes and a sample volume of about 2 μL [14]. This method has the potential for non-invasive early cancer detection and screening.

For the purpose of diagnosing cancer, Otieno et al. created a microfluidic immune-array technology to detect parathyroid hormone-related peptides (PTHrP) [15]. PTHrP has been associated with cancer metastasis in various human cancers and plays a significant role in humoral hypercalcemia of malignancy (HHM) [15]. The scientists used magnetic beads with enzyme labels and peptide-specific antibodies to create an ultrasensitive multiplexed peptide test to detect PTHrP 1-173. The device achieved an outstanding detection limit of 150 aM in a 30-minute timeframe utilizing a 5 μL sample. To characterize the electrical and mechanical characteristics of individual cancer cells, Zhou et al. created a microfluidic device; they investigated the deformability of Michigan Cancer Foundation- 7 (MCF-7) breast cancer cells by monitoring the time it takes a cell to pass through a narrow constriction [16]. By measuring the time it takes for cells to deform and pass through a narrow constriction, the device characterizes cell deformability and surface friction. Electrical impedance spectroscopy was used to analyze both undeformed and deformed cells. Combining mechanical and electrical properties provides a comprehensive set of intrinsic cellular biomarkers for improved differentiation of cellular phenotypes [16]. In order to get a high capture ratio for LNCaP-C4-2 prostate cancer cells, Ren et al. designed a microfluidic device that required five or six rows of micro constriction channels [17]. A sequential size-based microfluidic chip was developed to capture circulating tumor cells (CTCs) in prostate cancer. The chip utilized a series of microfiltration steps with gradually decreasing pore sizes to trap CTCs based on size. The captured CTCs were then analyzed and characterized. This approach demonstrated high capture efficiency and selectivity for CTCs, allowing for their isolation from blood samples [17].

Zielke et al. developed a droplet microfluidic technique called Sorting by Interfacial Tension (SIFT) for identifying cancer cell subpopulations based on glycolytic activity. High glycolysis rates in tumors have been associated with cancer metastasis, relapse, and poor prognosis. Identifying and targeting cells with high glycolysis levels may thus be critical for effective cancer therapy. The researchers successfully demonstrated that their platform is a reliable and straightforward method for isolating cancer cells with high glycolytic activity, which is crucial for researching particular therapeutic targets [18].

Malhotra et al. created an electrochemical microfluidic array for measuring four-protein panels to diagnose oral cancer. The instrument displayed an ultralow detection range of IL-6, IL-8, vascular endothelial growth factor, and vascular endothelial growth factor-C in serum [19]. The device utilized off-line protein capture with magnetic beads and demonstrated good correlation with traditional enzyme-linked immunosorbent assays. Serum analysis of oral cancer patients and controls yielded a clinical sensitivity of 89% and specificity of 98% [19]. The immunoarray is inexpensive, simple to make, and a useful test for diagnosis and personalized therapy of oral cancer (Table 1).

**Table 1:** Summary of Microfluidic Studies in Cancer Screening and Diagnostics.

Study	Objective	Method	Key Findings
Shah et al. [12]	Cell recovery using a biopolymer system	Affinity-based microfluidic device	- Captured and released cancer cells expressing EpCAM - Cell release process had 99% efficiency - Viability of released cells: 98.9% compared to control cells (99.4%) - Released cells showed 69.3% proliferation capacity
Zhao et al. [13]	Replicating metastasis in 3D cell culture system	Microwell array microfluidic devices	- Pairing tumor and stromal cell spheroids accurately - Analyzing 3D tumor invasion using high-content imaging
Chu et al. [14]	Multiplex-miRNA detection for cancer diagnostics	Nanomaterial-based microfluidic biochips	- Detected miRNA biomarkers in breast cancer with high accuracy - Detection time: ~35 minutes, sample volume: ~2 μL
Otieno et al. [15]	Microfluidic immune-array technology for PTHrP detection	Magnetic beads with enzyme labels and peptide-specific antibodies	- Detected PTHrP 1-173 with outstanding sensitivity (limit of detection: 150 aM) - Ultrasensitive multiplexed peptide test
Zhou et al. [16]	Characterizing electrical and mechanical properties of cancer cells	Microfluidic device	- Investigated deformability of MCF-7 breast cancer cells - Measured cell deformability and surface friction using narrow constrictions
Ren et al. [17]	Capture and characterization of circulating tumor cells (CTCs)	Size-based microfluidic chip	- Sequential microfiltration steps to capture CTCs based on size - High capture efficiency and selectivity for CTCs
Zielke et al. [18]	Identifying cancer cell subpopulations based on glycolytic activity	Droplet microfluidic technique (SIFT)	- Reliable and straightforward method for isolating cancer cells with high glycolytic activity - Useful for research on therapeutic targets
Malhotra et al. [19]	Electrochemical microfluidic array for oral cancer diagnosis	Off-line protein capture with magnetic beads	- Ultralow detection range for IL-6, IL-8, vascular endothelial growth factor, and vascular endothelial growth factor-C - Clinical sensitivity of 89% and specificity of 98% in oral cancer diagnosis

Artificial intelligence

Artificial Intelligence (AI) shows significant potential in revolutionizing cancer screening, diagnosis, and therapy by utilizing machine learning (ML) techniques that automate laborious tasks and analyze enormous amounts of data. Specifically, Deep Learning (DL), a subset of ML, has proven effective in detecting and segmenting cancerous lesions in various oncology applications. AI models have proven great accuracy in cancer screening, diagnosis, prediction, classification, and molecular marker identification, particularly in breast and colon cancer [4]. Furthermore, AI and Radiomics have improved glioma grading, surgical planning, and postoperative surveillance in CNS malignancies. Furthermore, AI has the potential to develop broadly applicable cancer screening systems for mammograms and colonoscopies, resulting in higher detection rates for many types of cancer [20].

Screening Mammogram

According to the National Cancer Institute, breast cancer is the most common cancer in the United States. AI has shown significant advantages in mammography screening. AI models have shown high accuracy compared to radiologists. However, when considering the same sensitivity level, the specificity of AI was lower than that of community-practice radiologists. But the combination of AI and radiologist assessment achieved higher accuracy in mammogram interpretation compared to AI or radiologists alone [21]. In studies of several DL models, sensitivity rates ranged from 88% to 96% overall [22]. Radiologists who used AI-assisted systems performed better in classification, performance, and sensitivity. AI-based Computer-Aided Design (CAD) systems have also improved sensitivity from 84% to 91% and decreased false positive rates by 69% [22]. To further improve detection accuracy and save effort by 70%, DL models are being included in digital breast tomosynthesis and contrast-enhanced digital mammography datasets for the volumetric evaluation of breasts in three dimensions [23].

Several recent studies have explored the potential of Computer-Aided Diagnosis (CAD) and Artificial Intelligence (AI) in the field of breast cancer [24]. One study introduced a Multiscale All Convolutional Neural Network (MA-CNN) for the classification of mammogram images, achieving promising results in automated breast cancer detection and classification [25]. These studies demonstrate the potential of CAD and AI technologies in improving diagnostic accuracy, aiding early detection, and providing valuable insights for personalized treatment strategies. In the United States, double readers are not common practice, but the potential for cost-effective AI in conjunction with radiologists may raise total sensitivity. AI also provides the benefit of reducing the time required for interpretation. To further increase accuracy rates, breast imaging has been subjected to radiomics, a technique to extract pertinent quantitative characteristics from clinical, histological, and radiological data [26].

Colonic Polyps and Colorectal Cancer

Colorectal cancer (CRC) is the third most prevalent cancer in the US, and artificial intelligence (AI) is being rapidly employed to enhance its detection, diagnosis, and care. AI algorithms have been developed to analyze historical data and complete blood counts (CBCs) to predict the likelihood of CRC and high-risk colonic polyps. Notably, two algorithms, ColonFlag and MeScore, have shown promising results in predicting polyps and CRCs based on CBC, demographic, and age data [27]. These models offer the potential for early and non-invasive screening, allowing for the identification of high-risk individuals who can be closely monitored [27].

AI is being used to improve the detection of colonic polyps during colonoscopy, which can help diagnose and screen for colon cancer. Computer-aided detection (CADe) and computer-aided diagnosis (CADx) systems have been developed to automate polyp identification during colonoscopy and provide further characterization of detected polyps [28]. An AI system known as Gastrointestinal (GI) Genius has demonstrated high detection rates and sensitivity for polyp identification, and real-time AI-aided colonoscopy has shown higher adenoma detection rates (ADR) compared to colonoscopy alone. By assisting endoscopists with higher ADR during screening colonoscopy, these AI-assisted algorithms can potentially reduce adenoma miss rates and the risk of post-colonoscopy CRC [29].

AI also plays a role in classifying colon polyps (CP) as malignant or non-malignant tumors. AI-assisted models have been developed to improve the accuracy and speed of CP diagnosis, which can be used with diagnostic techniques such as CT colonography and capsular endoscopy [30]. Deep learning (DL) models are utilized to accurately segment and define tumors, enabling faster and more precise identification of CRC metastases. With the introduction of AI-based algorithms for automating image processing, pathologists can now identify CPs with an average accuracy of 95% or higher [31]. Additionally, AI models have been employed to identify gene expressions, gene profiling, and non-coding micro-ribonucleotides (mi-RNAs) for targeted treatment planning, prognosis, and diagnosis. The use of AI in identifying microRNAs aids in the diagnosis, prognosis, and targeted therapy of CRC [32].

In summary, AI-based models have the potential to aid in the early detection of CRC by isolating circulating tumor cells and analyzing serum-specific biomarkers. These models can greatly assist pathologists in accurately classifying CPs and managing patients with CRC (Table 2).

**Table 2:** Applications of Artificial Intelligence (AI) in Breast and Colorectal Cancer Screening.

Application	Description
Mammography Screening	AI models have shown high accuracy in mammography screening, achieving sensitivity rates ranging from 88% to 96%. Combining AI with radiologist assessment improves accuracy and sensitivity. AI-based Computer-Aided Design (CAD) systems have also improved detection accuracy and reduced false positive rates.
Colonic Polyps and CRC	AI algorithms analyze historical data and complete blood counts (CBCs) to predict the likelihood of colorectal cancer (CRC) and high-risk colonic polyps. AI is used for polyp identification during colonoscopy, providing further characterization. Real-time AI-aided colonoscopy improves adenoma detection rates (ADR) and reduces adenoma miss rates.
Colonic Polyps Classification	AI-assisted models improve the accuracy and speed of colon polyp (CP) diagnosis, enabling faster and more precise identification of CRC metastases. AI-based algorithms automate image processing and gene profiling for targeted treatment planning, prognosis, and diagnosis of CRC.

Central Nervous System Cancers

The diagnosis and treatment of central nervous system (CNS) malignancies have been proven to benefit from the application of artificial intelligence (AI), notably in identifying and categorizing brain tumors. The gold standard technique for tumor identification and characterization right now is MRI, however, traditional approaches have drawbacks, such as a high chance of missing tumor foci infiltration [33]. By automating these procedures, AI has improved radiologists' efficiency and detection rates while reducing the time typically required for diagnosis. Additionally, Convolutional Neural Networks (CNN) based DL can identify Glioblastoma Multiforme (GBM) from metastatic brain lesions and find millimeter-sized brain tumors. Even with AI systems, it is still difficult to distinguish low-grade gliomas from high-grade gliomas on imaging. For the first time, attention-based transformers are being studied in categorizing gliomas, and their use might result in a breakthrough [34].

Initial observation, grading, determining the level of infiltration, segmenting and locating the tumor, histological analysis, and the discovery of molecular markers are all steps in the clinical care of central nervous system (CNS) malignancies. Clinical professionals create a treatment plan by manually assembling all the data for validation. AI has been proven to be helpful in this area, especially in improving radiologists' efficiency and detection rates. Although tumor distinction is usually predicted on histological examination, which is invasive, time-consuming, and costly, MRI technologies give organized anatomical information on tumors [35]. AI can recognize chemical compounds that are helpful for glioma grading and detecting tumor infiltrating locations, increasing the likelihood of discovery. Attention-based transformers are being researched for their potential to provide a breakthrough, although differentiating low-grade gliomas from high-grade gliomas on imaging remains difficult.

Radiomics

Clinical, histological, and radiological data are combined with machine learning and deep learning image processing in the emerging discipline of radiomics in neuro-oncology. This enables improved non-invasive tumor characterization and prognostication, monitoring, and therapy response assessment. The two primary algorithms used in radiomics are feature-based and DL-based, and both provide outcomes that are more accurate and trustworthy than those of readers [20]. While DL radiomics employ CNN to learn a cascade manner without previous feature definition, feature-based algorithms analyze subsets of particular features from segmented areas and volumes of interest. Imaging pre-processing, tumor segmentation, feature extraction, feature selection, model creation, and model assessment are all steps in the complicated multi-step process of radiology [26].

Histopathological Aspects, Genetics, and Molecular Marker Detection

It may be difficult to accurately diagnose cranial tumors histopathologically, which might produce false-positive findings. Microscope slides are now digitized, and AI-based algorithms like support vector machines (SVM) and decision trees are utilized to analyze malignant glioma specimens and forecast outcomes based on genetic and molecular markers [36]. Isocitrate dehydrogenase (IDH) mutation status, 1 p/19 co-deletion status, Methylguanine-DNA Methyltransferase (MGMT) methylation status, epidermal growth factor receptor splice variant III (EGFRvIII), Ki-67 marker expression, and prediction of BRAF and catenin -1 mutations are some of these indicators. In order to predict MGMT methylation status with up to 83% accuracy, AI can identify these biomarkers from traditional MRI modalities and apply principal component analysis to the last layer of CNN [37]. As an alternative to the invasive and time-consuming conventional technique of detection through immunohistochemistry tests on the removed tumor sample, AI-based radiomics has also been developed to identify Ki-67 marker expression from fluorodeoxyglucose (FDG)-positron emission tomography (PET) and MRI images [38].

Preoperative Assessment

Planning therapy for CNS tumors requires precise segmentation, volumetric evaluation, and tumor differentiation from normal brain tissue and peripheral edema. The tumor has been precisely and accurately localized by applying AI algorithms like CNN and SVM to the tumor parts. The automatic segmentation of gliomas using 3D-U-Net CNN on 18-fluoroethyl-tyrosine-PET has shown excellent results [39].

Intraoperative Modalities

High-grade tumors, like GBM, proliferate rapidly and spread beyond the augmenting areas seen on radiographs. AI-based deep learning algorithms now enable surgeons to excise a greater amount of tumors while preserving healthy brain tissue. Differentiating primary brain tumors, main CNS lymphoma, and brain metastases has been accomplished using decision trees and multivariate logistic regression models [40].

Postoperative Surveillance

The gold standard for assessing postoperative tumor development and tumor response is MRI with gadolinium contrast. Clinical, imaging, genetic, and molecular marker data have been used to predict treatment response and survival outcomes using AI-based algorithms like CNN and SVM. CNN models can distinguish between real and fake progression, while ML algorithms can distinguish between radiation necrosis and tumor recurrence [41].

Precision and Personalized Medicine

Through the use of clinical decision support systems, chemotherapy, immunotherapy, and radiation treatment, AI has made it possible to practice precision and personalized oncology. A more focused approach to treatment is possible because of the development of machine learning (ML) algorithms that can forecast the effects of chemotherapy medications based on genetic fingerprints and identify cancer cells with HR abnormalities. For example, in CRC, PI3K alpha and tankyrase have been identified as promising therapeutic targets using DL algorithms [40]. Big data from the clinical setting combined with AI may be used to create personalized treatment plans for patients based on various variables. AI can synthesize and evaluate enormous volumes of chemical data to design novel cancer treatments. AI may also assist radiologists in scheduling radiation treatments and anticipating the therapeutic benefits of immunotherapy [37]. By offering better, more individualized treatment alternatives that are also time-effective, AI has thereby transformed oncology.

Biosensors for biomarker detection

As an accurate, quick, and sensitive analytical test, biosensors are utilized to identify cancer biomarkers. To find cancer biomarkers and transform biological signals into observable electrical or visual forms, they use certain biomolecules as biorecognition components. According to the transduction principle, biomarkers may be classified as electrochemical, mass-sensitive, or optical. Due to their sensitivity, specificity, affordability, and potential for miniaturization, electrochemical biosensors (EB) are chosen [42]. Cancer biomarkers are found using biorecognition components such as proteins, Deoxyribonucleic acid (DNA), enzymes, and aptamers. EBs may be divided into immunosensors, apta-sensors, enzymatic biosensors, and nucleic acid biosensors based on the biorecognition element used [42].

Nucleic Acid-Based Biomarker Detection

Due to their sensitivity in detecting minute oligonucleotide concentrations, single-base mismatches, and straightforward construction, nucleic acid-based biosensors are employed to quickly and precisely detect cancer biomarkers. These indicators make identifying cancer in people with no visible symptoms possible. Detecting nucleic acid-based cancer biomarkers has been suggested using various electrochemical techniques. Using a magnetically controlled EB with a limit of detection (LOD) as low as 2.2 × 10−19 M, Zhang et al. made it possible to diagnose oral cancer instantly [43]. Based on biotinylated complementary probes immobilization on magnetic beads (MBs) coated with streptavidin, Boriachek et al. presented an EB for detecting miRNA from human serum. Differential pulse voltammetry (DPV) was used to measure the electrochemical response with a LOD of 1.0 pmol/L [44]. With a LOD of 2.3 fM, Luo et al. created a locked nucleic acid-based EB for exosomal miRNA-21r detection. Electrochemical impedance spectroscopy (EIS) and differential pulse voltammetry (DPV) were used to verify the sensor [45]. Recently, a biosensor made of single-walled carbon nanotubes and fluorine-doped tin oxide was suggested for detecting miRNA-21, a particular biomarker for various cancer types with a LOD as low as 0.01 fmol L1.

Protein/Immune-Based Biomarker Detection

Advancements in analytical instruments have made protein biomarkers an important target for cancer detection. The discovery of protein-based cancer biomarkers in biological samples has led to the development of several biosensors. Epidermal growth factor receptor (EGFR) detection limits of 0.88 pg/mL and 0.34 pg/mL, respectively, were recommended by Elshafey et al. for use in human plasma and phosphate buffer [46]. Carcinoembryonic antigen (CEA) has a good detection limit of 1 104 ng mL1, and Zhang et al. created a nanocomposite-based electro-sensing platform for its detection [47]. An immune-sensing device with a detection limit of 5.3 pg/mL for CEA was suggested by Luo et al. and is based on single-walled carbon nanotubes (SWCNTs), quantum dots, and reduced graphene oxide- gold nanoparticles (AuNPs) [45]. To detect HER3, with a linear detection range of 0.2-1.4 pg/mL, Canbaz et al. covalently bonded the complementary HER3 Ab to a nanomodified gold electrode [48].

The breast cancer biomarker HER2- extracellular domain (ECD) was identified in human blood with a LOD of 2.1 ng/mL in another investigation using a disposable EB. By analyzing a number of human proteins and another cancer biomarker, cancer antigen (CA) 15-3, the stated sensor's sensitivity was verified. For the detection of epidermal growth factor receptor (EGFR) with a LOD of 50 pg/mL, Ilkhani et al. developed an aptamer-based biosensing assay. Biotinylated EGFR aptamer immobilized on streptavidin-modified graphene oxide served as the foundation for the biosensor [49]. These biosensors provide doctors with a sensitive and dependable tool for spotting cancer even in its earliest stages.

Electrochemical Apta-sensors for Cancer Biomarker Detection

The detection of cancer biomarkers is a common use for electrochemical apta-sensors. Three categories of electrochemical apta-sensors for cancer biomarkers exist, including those that recognize exosomes, circulating tumor cells, and protein tumor biomarkers. In one work, microgel nanocomposites were used to create a highly sensitive electrochemical apta-sensor for the detection of miRNA-21. With a linear range of 10 aM to 1 pM, the apta-sensor displayed a low LOD of 1.35 aM. For the simultaneous detection of carcinoembryonic antigen (CEA) and cancer antigen 15-3 (CA 15-3) in blood samples, another apta-sensor was created [50]. This apta-sensor demonstrated LODs of 11.2 pg mL-1 and 11.2 x10-2 U mL-1 for CEA and CA 15-3, respectively. It employed a nanocomposite of gold nanoparticles (AuNPs) and 3D graphene hydrogel.

Another research used the LC-18 aptamer to create an electrochemical apta-sensor for detecting lung cancer biomarkers. The immobilization of thiolated aptamer on gold disc electrodes created the apta-sensor, demonstrating high specificity for lung cancer-related proteins and cells. Additionally, a label-free electrochemical apta-sensor for the detection of CA-125, a marker for the diagnosis of ovarian cancer, was created utilizing nickel hexacyanoferrate (NiHCF) nanocubes and polydopamine-functionalized graphene (PDA/GR) [51]. With a linear range of 0.10 pg mL-1 to 1.0 g mL-1, this apta-sensor showed a low LOD of 0.076 pg mL-1.

Biosensors for Diagnosing Multiple Biomarkers

Since no single biomarker is sufficiently specific for each kind of cancer, there is rising interest in creating multi-biomarker platforms for cancer detection, according to a number of studies. To detect many cancer indicators with high sensitivity and specificity, researchers have created a variety of biosensors. For instance, Chen et al. developed an electrochemical sandwich platform based on bio-functional carboxyl graphene nanosheets (CGS) to detect CEA and alpha-fetoprotein (AFP) with respective limits of detection of 0.1 ng/mL and 0.05 ng/mL [52]. Similarly, Atlintas and his coworkers created a biosensor that can track CEA and EGFR over a linear range of 20-1000 pg/mL and detect CA15-3 over a broad range of 10-200 U/mL [53]. With detection limits of 0.23 pg/mL and 0.30 pg/mL, respectively, Malhotra et al. developed an EI capable of detecting two prostate cancer biomarkers, Prostate-specific antigen (PSA) and interleukin (IL) 6 [19].

With LODs of 0.7 pg/mL, 0.007 U/mL, and 0.9 pg/mL, respectively, Hong and coworkers disclosed a gold-modified indium tin oxide (ITO) electrode that could detect CEA, CA125, and PSA [54]. With LODs of 0.8 pg/mL, 0.005 U/mL, and 0.7 pg/mL, respectively, biotin-doped polypyrrole was also created to detect CEA, CA125, and PSA cancer biomarkers.

Additionally, scientists have created a number of biosensors that can simultaneously detect multiple cancer biomarkers. Wilson and Nie have developed a second biosensor for the detection of seven cancer biomarkers connected to different types of cancer, including CEA, human chorionic gonadotropin (hCG), alpha-fetoprotein (AFP), CA125, CA15-3, ferritin, and CA19-9. The development of efficient cancer screening technologies is made possible by the high potential for early and accurate cancer detection offered by these biosensors [55].

## Conclusions

Cancer screening and diagnosis have advanced significantly with the development of new technologies and techniques, such as microfluidic devices, non-invasive procedures, and machine learning algorithms. These advancements have enabled early cancer detection, improving the chances of effective treatment and management. In particular, biomarker-based cancer diagnosis utilizing biosensing devices with different nanoparticles offers promising potential for early detection and disease progression monitoring. Overall, the use of advanced technologies and techniques in cancer screening and diagnosis has the potential to improve patient outcomes greatly, and continued research and development in this field is critical.
